# Diagnostic performance of 3T MRI preoperative localization of parathyroid adenomas in primary hyperparathyroidism

**DOI:** 10.3389/fendo.2026.1862421

**Published:** 2026-08-20

**Authors:** Lukáš Lambert, Ivan Raška, Václav Hána, Monika Wagnerová, Andrej Bocán, Jan Hrdlička, Judita Klímová, Petr Libanský, Dana Michalská, Kateřina Zajíčková, Aleš Antonín Kuběna, Andrea Burgetová, Vít Zikán

**Affiliations:** 1 Department of Radiology, General University Hospital and 1^st^ Faculty of Medicine, Charles University, Prague, Czechia; 2 Department of Imaging Methods, Motol University Hospital and 2^nd^ Faculty of Medicine, Charles University, Prague, Czechia; 3 Faculty of Health Studies, Technical University Liberec, Liberec, Czechia; 4 Third Department of Internal Medicine, General University Hospital and 1^st^ Faculty of Medicine, Charles University, Prague, Czechia; 5 Institute of Nuclear Medicine, General University Hospital and 1^st^ Faculty of Medicine, Charles University, Prague, Czechia; 6 Department of Medicine, 1^st^ Faculty of Medicine, Charles University and Military University Hospital in Prague, Prague, Czechia; 7 Third Department of Surgery, Motol University Hospital, Charles University, Prague, Czechia; 8 Institute of Endocrinology, Prague, Czechia; 9 Institute of Medical Biochemistry and Laboratory Diagnostics, General University Hospital and 1^st^ Faculty of Medicine, Charles University, Prague, Czechia

**Keywords:** dynamic contrast enhancement (DCE), magnetic resonance imaging, multi-gland disease, parathyroid adenoma, primary hyperparathyroidism, single gland disease

## Abstract

**Objective:**

Preoperative localization is essential for the surgical management of primary hyperparathyroidism (PHPT). Ultrasound and 99mTc-sestamibi SPECT/CT are first-line imaging modalities, while MRI’s role is less defined. This study aimed to evaluate MRI for parathyroid adenoma localization in PHPT and compare it with that of ultrasound and SPECT/CT.

**Methods:**

In this prospective single-center study, 103 adults with biochemically confirmed PHPT underwent ultrasound, multiparametric 3T MRI, and SPECT/CT before surgery. MRI examinations were independently evaluated independently evaluated by two blinded radiologists. Surgical and histopathological findings served as the reference standard.

**Results:**

Ninety-three patients (90%) had single-gland disease (SGD), and 10 (10%) multiglandular disease (MGD). MRI demonstrated high sensitivity for adenomas in SGD but significantly lower sensitivity in MGD: Reader 1, 0.824 vs 0.476 (p = 0.002) and Reader 2, 0.890 vs 0.524 (p < 0.001). Ultrasound and SPECT/CT showed similar patterns. Specificity did not differ significantly between SGD and MGD. Inter-reader agreement was high for MRI and ultrasound (Krippendorff’s α > 0.8) but decreased when including SPECT/CT. MRI-based size lesion size measurements showed good concordance between readers (ρC = 0.822) but showed low concordance with histology (ρC1 = 0.498; ρC2 = 0.373) due to systematic underestimation (15–18%).

**Conclusion:**

Multiparametric 3T MRI provides localization sensitivity to that of first-line imaging modalities in SGD, but differentiation between MGD and SGD remains challenging, with MRI showing no clear superiority over standard modalities.

## Introduction

Primary hyperparathyroidism (PHPT) is the third most common endocrine disorder (after diabetes and thyroid disease) characterized excessive or inappropriately normal parathyroid hormone secretion, usually resulting in hypercalcemia (1). Clinical manifestations of PHPT may include skeletal involvement, such as reduced bone mineral density and increased fracture risk, nephrolithiasis, nephrocalcinosis, gastrointestinal symptoms, and neuropsychiatric symptoms (2). PHPT is mostly caused by a solitary benign parathyroid adenoma (single-glandular disease, SGD) in 85-90%, followed by multiglandular disease in 5%–10% (MGD), and, rarely, parathyroid carcinoma (<1%) (3, 4).

The diagnosis of PHPT is biochemical; imaging is performed after a decision to proceed with surgery to localize the abnormal parathyroid gland(s) and guide the operative approach (2). The only curative treatment is parathyroid surgery (2, 5). Preoperative localization of parathyroid adenoma (PA) by imaging is therefore essential to achieve favorable surgical outcome while minimizing invasiveness. Ultrasound (US) and SPECT/CT with ^99m^Tc-sestamibi (MIBI)/^123^Iodine scintigraphy are the first line imaging methods (6). Second line imaging methods that can further improve localization include four-dimensional CT (4D CT), MRI, and ^18^F-fluorocholine PET/CT (6, 7).

Although ultrasound and sestamibi scintigraphy remain first-line imaging modalities according to current guidelines, ^18^F-fluorocholine PET/CT has become the preferred second-line examination in many high-volume endocrine surgery centers because of its excellent diagnostic performance, particularly in patients with negative or discordant first-line imaging. Despite its high diagnostic performance in most of the patients use of ^18^F-fluorocholine PET/CT bears limitations such as radiation, use of iodine contrast for better resolution, high price and limited availability. 3T MRI overcomes these limits in situations like PHPT in pregnancy, patients allergic to iodine, regions with restrictive reimbursement costs for PET imaging, cases where enhanced spatial resolution for consequent surgery is desired.

The detection of PA on MRI is based on high signal intensity on T2-weighted images and early arterial enhancement with gradual washout (8). PAs have homogeneous appearance, are elongated in craniocaudal direction and do not exhibit restricted diffusion (8, 9). While several studies have evaluated the utility of MRI in the detection of PAs, they were retrospective, not blinded, or comprised smaller numbers of patients (10, 11).

The aim of this prospective study was to assess the diagnostic performance of multiparametric 3-T MRI for localizing of parathyroid adenomas in PHPT and to compare its performance with that of other imaging modalities.

## Materials and methods

This prospective study was conducted in accordance with the Declaration of Helsinki and approved by the Ethics Committee of the General University Hospital in Prague 16.2.2023 under No. 11/23 grant GIP. Written informed consent was obtained from all participants prior to inclusion, MRI imaging and data collection.

Consecutive patients diagnosed with primary hyperparathyroidism between January 2022 and December 2024 were included in the study. The inclusion criteria were primary biochemical diagnosis of PHPT according to the diagnostic criteria of the European Society of Endocrinology (2); age ≥ 18 years; consent to undergo MRI with administration of contrast material; written informed consent. Exclusion criteria were CKD stages 4-5, hereditary or genetic PHPT, previous parathyroid surgery.

**Figure 1:**
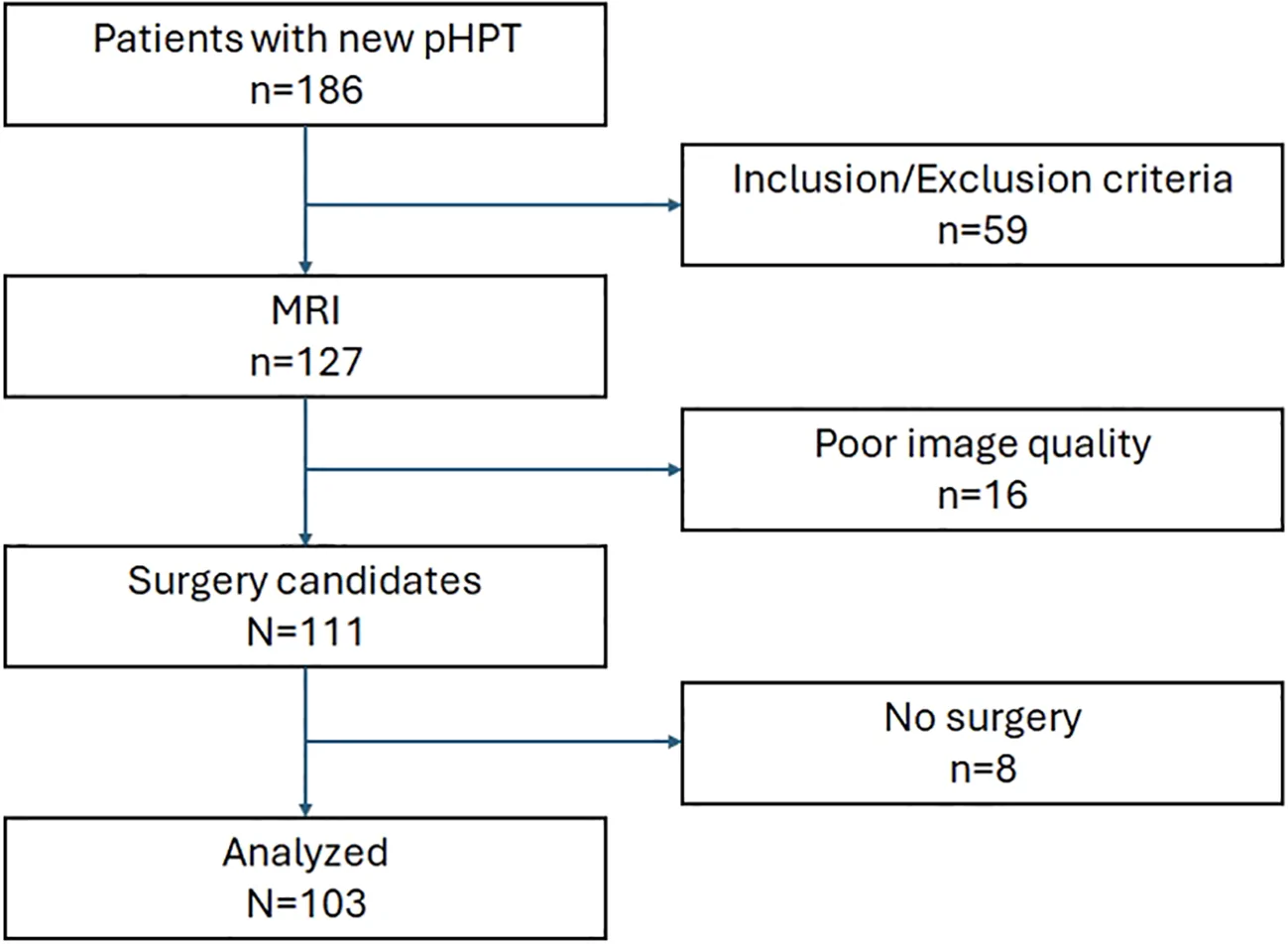
Study flowchart.

From 186 patients with newly diagnosed PHPT, 127 patients met the inclusion criteria and were enrolled, from which 16 were excluded due to poor image quality on MRI, 8 did not undergo surgery (refused after localization imaging), resulting in 103 patients who were ultimately analyzed (**Figure 1**; **Table 1**).

**Table 1:** Patient characteristics.

Parameter	All patients n=103	SGD n=93	MGD n=10	P value
Females (number)	86 (83%)	79 (85%)	7 (70%)	0.802
Age (years)	64 (55-69)	63 (54-69)	70 (60-75)	0.060
Height (cm)	167 ± 8	167 ± 8	168 ± 6	0.629
Body mass index (kg/m^2^)	27.3 (23.9-30.2)	27.1 (23.8-30.4)	27.5 (23.4-30.9)	0.844
Total calcium (mmol/l)	2.78 (2.65-2.91)	2.78 (2.66-2.92)	2.75 (2.63-2.84)	0.518
Ionized calcium (mmol/l)	1.37 (1.21-1.45)	1.37 (1.32-1.45)	1.33 (1.30-1.41)	0.358
Phosphate (mmol/l)	0.82 ± 0.15	0.81 ± 0.15	0.83 ± 0.15	0.616
Parathormone (pmol/l)	10.8 (8.5-14.4)	10.8 (8.6-14.2)	10.7 (7.7-15.8)	0.824
25-OHD (ng/ml)	23.0 ± 8.4	22.6 ± 8.5	26.8 ± 6.0	0.130
Creatinine (µmol/l)	71 (60-81)	70 (60-80)	78 (70-86)	0.086
ALP (µkat/l)	1.60 (1.38-2.07)	1.61 (1.39-2.08)	1.51 (1.38-2.07)	0.722

25-OHD, 25-hydroxyvitamin D; ALP, alkaline phosphatase. Values presented as mean ± SD or median (with quartile range), or number (%).

### Biochemical analysis

After a 12-hour overnight fast, venous blood samples were obtained from all participants. Routine biochemical analyses were conducted using fresh specimens. Serum levels of calcium, phosphate, creatinine, alkaline phosphatase (ALP), and parathyroid hormone (PTH) were measured using standardized automated analytical methods (Modular, Roche Diagnostics, Germany) (2). 25(OH)D was determined by CLIA (DiaSorin Inc).

### Ultrasound

Ultrasound of the neck was performed by a board-certified endocrinologist using a linear probe (4-12MHz) and a high-end ultrasound machine (GE Logiq E10, GE Healthcare, Chicago, IL) as a first-line imaging method. The scanning extended from the mouth floor to the jugulum. Parathyroid glands were identified on ultrasound as well-defined, homogeneously hypoechoic, oval structures with variably increased vascularity on color Doppler supporting hyperfunctioning tissue. The largest dimension was measured using electronic callipers.

### Magnetic resonance imaging

**Figure 2:**
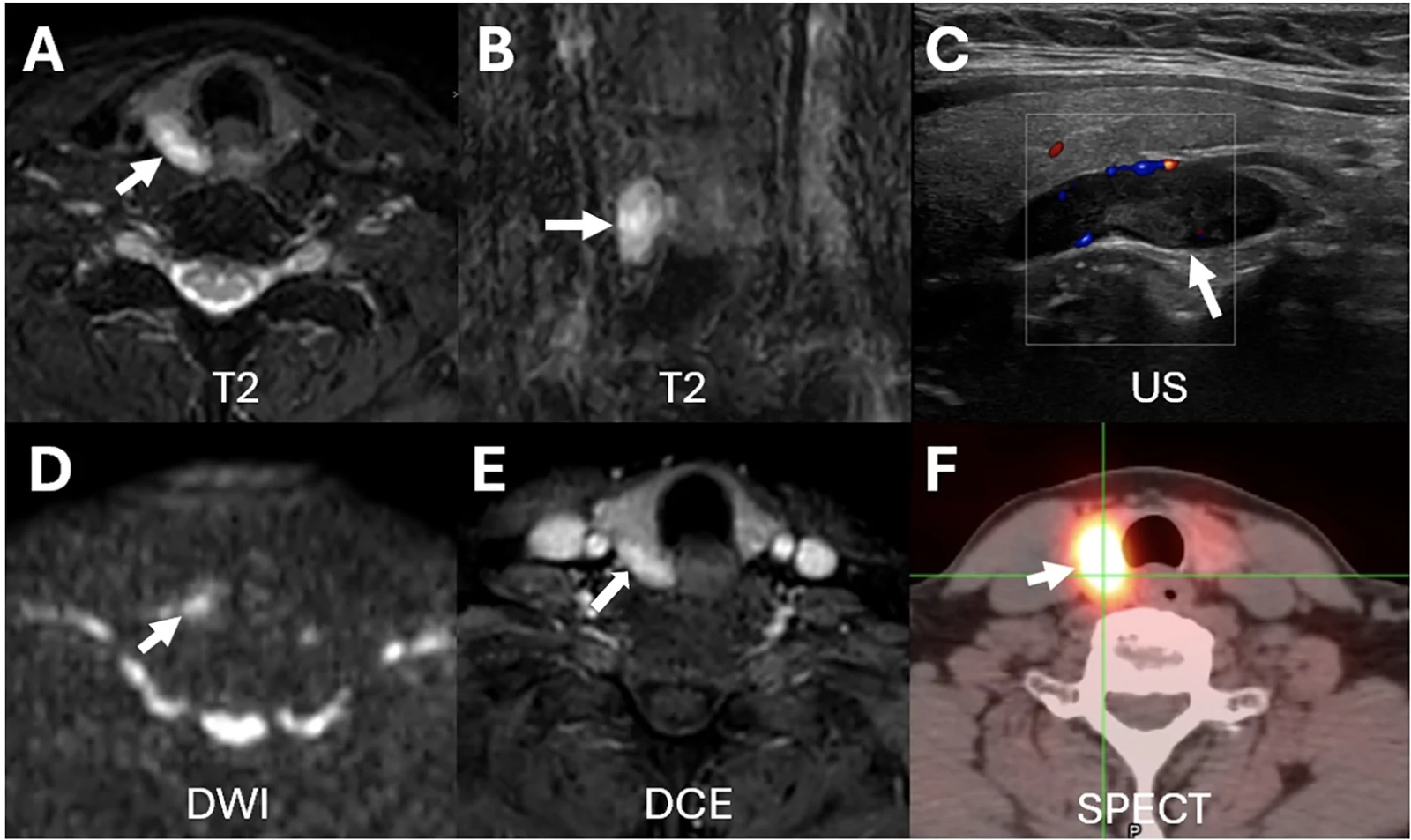
Right upper parathyroid adenoma on MRI in T2 weighted DIXON **(A, B)**, diffusion weighted imaging **(D)**, dynamic contrast enhancement **(E)**, on ultrasound [US, **(C)**], and single-photon emission tomography SPECT/CT, **(F)**].

MRI was performed on a 3T MRI scanner (Ingenia Elition 3T, Philips, Best, The Netherlands) during free breathing using a head-neck coil with the following sequences: T2-weighted DIXON (transverse, coronal, sagittal planes), diffusion-weighted imaging (DWI, transverse), and vymazat T1 weighted fat saturated dynamic sequence with intravenous administration of Gd-based contrast material (T1-free-breathing) (**Supplementary Table 1**). The examination extended from the mouth floor to the upper mediastinum. The images were analyzed on an enterprise workstation (Intellispace, Philips, Best, The Netherlands) by two board-certified radiologists (Reader 1 and Reader 2). Both radiologists were blinded to clinical, biochemical, ultrasound, and nuclear medicine findings. Image quality assessment (motion artifacts, susceptibility artifacts, incomplete fat suppression) was performed prior to interpretation by one radiologist (Reader 1). The typical appearance of PA was homogeneously high signal of T2 fat saturated, elongated morphology, early enhancement (**Figure 2**) (7, 8). A distinction from thyroid nodule was also based on the presence of intervening fat plane.

### SPECT/CT ^99m^Tc-sestamibi scintigraphy

Patients fasted for at least 4 hours prior to imaging. Each received intravenous ^99m^Tc-pertechnetate for thyroid imaging (200MBq in 70 kg male) followed by ^99m^Tc-sestamibi (700MBq in 70 kg male) for parathyroid imaging. Early planar images were acquired 10–15 minutes post-sestamibi injection, with delayed planar and SPECT/CT imaging at 90–150 minutes using a GE Infinia Hawkeye 4 (GE Healthcare, Chicago, IL, USA). Subtraction of thyroid activity on fused images served for localization of hyperfunctioning parathyroid tissue. The images were evaluated by a nuclear medicine physician (M.W.). The extent of the examination was from the skull base to the diaphragm.

### 18F-cholin PET/CT

In patients where at least two of the three methods (MRI, ultrasound, SPECT) were inconclusive ^18^F-fluorocholine PET/CT was performed using a GE Discovery 690 PET/CT system (GE Healthcare, Chicago, IL, USA). Images were acquired approximately 60 minutes after intravenous injection of ^18^F-fluorocholine (activity 2–4 MBq/kg) and reconstructed with iterative algorithms for attenuation correction and anatomical localization.

### Surgery

Parathyroidectomy was performed by a surgical team routinely performing parathyroid surgery. Surgical localization and postoperative histopathological confirmation of parathyroid adenoma were used as the gold standard for adenoma localization. Postoperative normalization of calcium and parathormone were used as a marker of successful surgery.

### Statistical analysis

Data analysis was performed using Wolfram Mathematica 13.2, R, and GraphPad Prism software. Descriptive statistics for quantitative variables were reported as mean ± standard deviation (SD) or median with quartiles (LQ, UQ), depending on the distribution assessed by the D’Agostino-Pearson omnibus test for normality. Dichotomic variables were summarized as counts and proportions (%). The sample size was calculated to estimate the sensitivity of MRI for the localization of parathyroid adenomas with sufficient statistical precision. Based on an expected sensitivity of 0.85 from prior literature, at least 77 patients with surgically confirmed SGD were required to achieve a two-sided 95% confidence interval with a predefined precision (half-width) of ±0.08. To account for anticipated exclusions and the expected prevalence of MGD, which affects localization sensitivity, the target enrollment was set at approximately 100 patients.

Per-lesion sensitivity for each imaging modality was calculated with 95% confidence intervals (CIs) derived using the Clopper-Pearson exact method. Differences in sensitivity and specificity between patients with MGD and SGD were evaluated using Fisher’s exact test due to the categorical nature of the data and the relatively small subgroup sizes. Inter-rater agreement for binary detection outcomes was assessed using Krippendorff’s alpha (α), selected for its suitability in handling multiple raters and binary data. According to established conventions, values of α greater than 0.8 were interpreted as indicating reliable agreement. Adenoma size measurements were log-transformed prior to analysis to address skewness in the size distribution and to ensure proportionality in comparisons across modalities. Agreement in size estimation between readers and between imaging modalities and histology was quantified using Lin’s concordance correlation coefficient (ρC), which decomposes concordance into components of precision and accuracy, providing insight into both variability and systematic bias. Bland-Altman plots on log-transformed data were employed to visualize agreement and assess the magnitude and direction of differences in size estimates between MRI, ultrasound, and histology. Systematic bias between MRI-based and histological adenoma size estimates was evaluated using the Wilcoxon signed rank test a non-parametric method for detecting consistent under- or overestimation in paired measurements.

## Results

A total of 103 patients who underwent surgical intervention were included in the final analysis (**Figure 1**; **Table 1**). All analyzed patients achieved postoperative normocalcemia, confirming the success of the surgical treatment.

**Figure 3:**
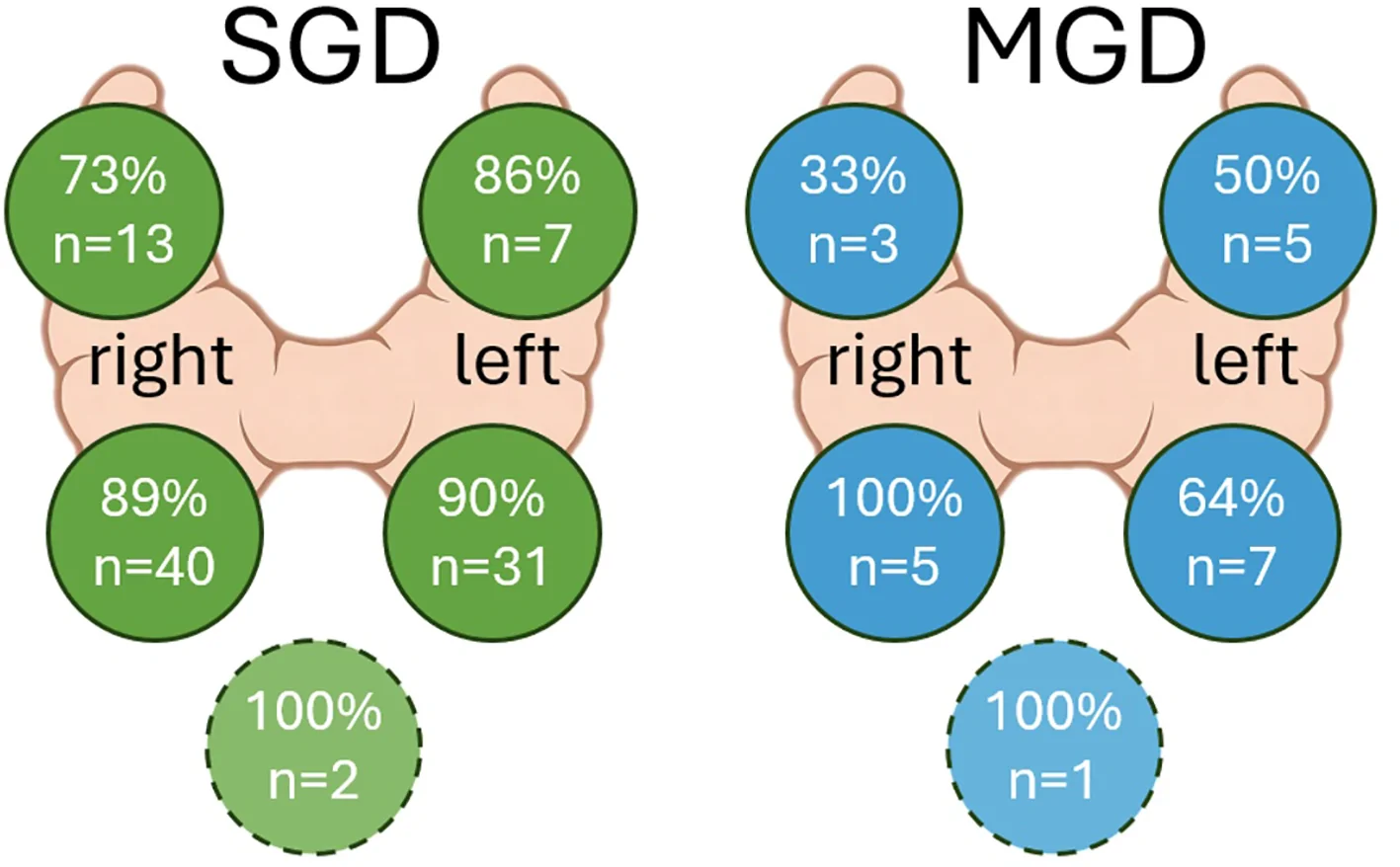
Number of parathyroid adenomas found on surgery (n) and MRI sensitivity in their detection on MRI (%, average from both readers) in single-gland (SGD) and multiglandular disease (MGD). Dashed circle denotes ectopic localization. Lesions in the jugulum and mediastinum were identified in SGD, and one jugulum lesion was correctly detected in MGD.

Histopathological examination identified a total of 115 adenomas, comprising 21 adenomas in 10 patients with MGD and 93 adenomas in patients with SGD. Among these, two adenomas in SGD patients and one adenoma in an MGD patient were located ectopically. MRI assessments by Reader 1 detected 10 orthotopic adenomas in MGD patients and 75 in SGD patients, while Reader 2 identified 11 and 81 orthotopic adenomas in these groups, respectively (**Figure 3**). Ultrasound detected 9 adenomas in MGD and 77 in SGD patients; however, ultrasound was not usable in 4 MGD and 2 SGD patients. MIBI scintigraphy identified 10 adenomas in MGD and 69 in SGD patients, with the procedure not performed in 2 MGD and 3 SGD patients. ^18^F-fluorocholine PET/CT was used in 17 patients, where it correctly identified 2/4 adenomas in 2 MGD patients and 15/15 adenomas in SGD patients. Lesion distribution (left-to-right and upper-to-lower) did not differ between visible and non-visible adenomas on MRI (p = 0.73 and p = 0.37). All three ectopic adenomas were detected by both MRI readers. Ultrasound detected two ectopic adenomas, both in SGD patients, while MIBI identified one ectopic adenoma in an MGD patient; notably, one ectopic adenoma identified by histology was in a patient who did not undergo MIBI.

Sensitivity estimates, including confidence intervals, are detailed in **Table 2**. Both MRI readers demonstrated significantly higher sensitivity for detecting orthotopic adenomas in SGD compared to MGD patients: Reader 1 achieved sensitivities of 0.824 versus 0.476 (p = 0.0017, Fisher exact test), and Reader 2 achieved 0.890 versus 0.524 (p < 0.001). Similarly, ultrasound sensitivity was 0.846 in SGD versus 0.429 in MGD (p = 0.0035), and MIBI sensitivity was 0.758 in SGD versus 0.476 in MGD (p = 0.040). Specificity did not differ significantly between SGD and MGD groups across any of the four methods (Reader 1: p = 0.293; Reader 2: p = 0.066; ultrasound: p = 0.533; MIBI: p = 0.267).

**Table 2:** Per-lesion sensitivity (95% CI) of imaging modalities for detection of parathyroid adenomas in the overall cohort, stratified by MGD and SGD, with patient-based detection accuracy (match/all).

Imaging modality	Per-lesion sensitivity (95% CI)	Diagnosis MGD vs. SGD	Patients match/all
MRI – reader 1	0.759 (0.669–0.835)	MGD: 0.476 (0.257–0.702) ^**^ SGD: 0.824 (0.730–0.896)	3/10 78/93
MRI – reader 2	0.821 (0.738–0.887)	MGD: 0.524 (0.298–0.743) ^***^ SGD: 0.890 (0.807–0.946)	3/10 84/93
Ultrasound	0.811 (0.679–0.842)	MGD: 0.429 (0.218–0.660) ^**^ SGD: 0.846 (0.755–0.913)	2/8 79/91
*^99m^Tc* MIBI	0.738 (0.612–0.788)	MGD: 0.476 (0.257–0.702) ^*^ SGD: 0.758 (0.657–0.842)	3/9 70/89

Per-lesion sensitivity is reported with 95% Clopper–Pearson confidence intervals (CI). Patient-based detection accuracy is expressed as match/all. Sensitivity differences between MGD and SGD were assessed using Fisher’s exact test (*p < 0.05; **p < 0.01; ***p < 0.001). Abbreviations: MGD, multiglandular disease; SGD, single-gland disease; CI, confidence interval; ^99m^Tc-MIBI, technetium-99m methoxyisobutylisonitrile.

Inter-reader agreement for detection of orthotopic adenomas (binary data) was high, with Krippendorff’s α exceeding 0.8 between the MRI readers and between the readers and ultrasound, indicating reliable agreement according to Krippendorff’s convention. This reliability persisted within both MGD and SGD subgroups and overall. Inclusion of MIBI reduced agreement slightly below the reliability threshold but remained tentative overall (α = 0.783) and within the SGD subgroup (α = 0.796). In the MGD subgroup, agreement decreased further (α = 0.659), falling below the reliability threshold. Detailed Krippendorff’s α values are presented in **Table 3**.

**Table 3:** Inter-rater and inter-modality reliability in lesion localization using a hierarchical (cascade) approach.

Comparison level	Overall	MGD group	SGD group
MRI 1 + 2	0.875	0.828	0.881
MRI 1 + 2 + Ultrasound	0.806	0.823	0.803
MRI 1 + 2 + Ultrasound + MIBI	0.783	0.659	0.796

Reliability was assessed using per-lesion Krippendorff’s α with hierarchical modality inclusion: Level 1, inter-rater MRI reliability; Level 2, agreement between MRI and ultrasonography; Level 3, consensus across all modalities including scintigraphy (MIBI). Abbreviations: MGD, multiglandular disease; SGD, single-gland disease; MIBI, technetium-99m methoxyisobutylisonitrile.

**Figure 4:**
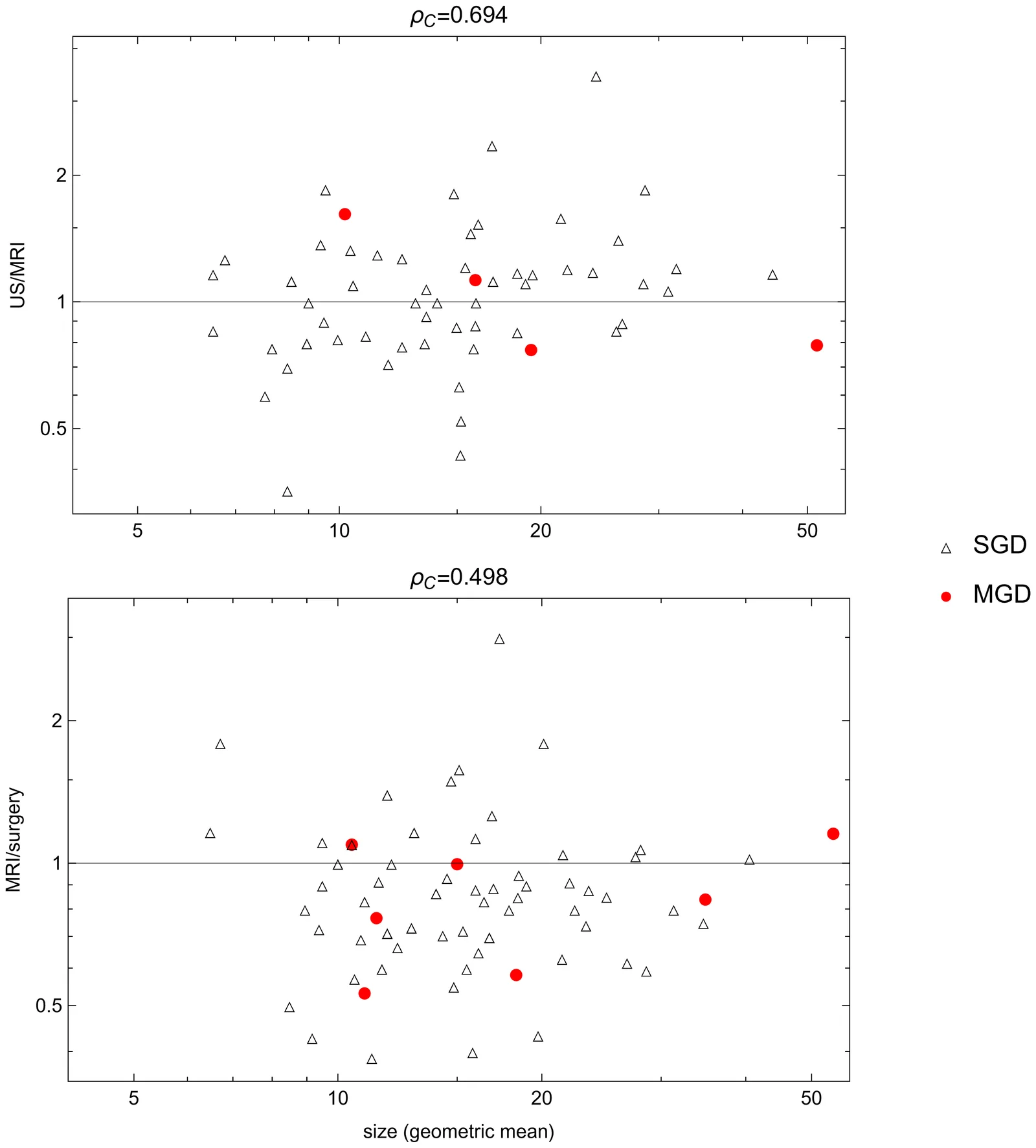
Bland-Altman log-log plots showing agreement in lesion size estimation between MRI and ultrasound (US) (top) and between MRI and surgical measurements (bottom). Agreement in maximal lesion dimensions (mm) is shown on log-log scales to account for proportional error. The x-axis represents the geometric mean of the compared measurements. Top panel: Ultrasonography (US) versus Magnetic Resonance imaging (MRI). Bottom panel: MRI versus post-operative histology (surgical specimen). The reference line ratio=1 (grey) represents the perfect agreement. Symbols p indicate overall Lin's concordance correlation coefficients.

The Lin’s concordance correlation coefficient (ρC) for the logarithmic estimates of adenoma size between Reader 1 and Reader 2 was 0.822 based on 87 paired assessments. Concordance with histological measurements was lower: ρC1 = 0.498 (78 pairs) for Reader 1 and ρC2 = 0.376 (N = 86 pairs) for Reader 2. Ultrasound measurements were comparable to MRI (median 14 mm with quartiles 9–20 mm, p = 0.65). Concordance between MRI and ultrasound size estimates was also reduced: ρ’C1 = 0.694 (N’1 = 67 pairs) for Reader 1 and ρ’C2 = 0.636 (N’2 = 76 pairs) for Reader 2. Bland-Altman log-log plots illustrating agreement between Reader 1 and ultrasound as well as histology are shown in **Figure 4**. The precision-accuracy decomposition of Lin’s concordance coefficients between MRI and histology revealed contributions from both bias and inaccuracy to the low concordance: Reader 1 had ρC1 = 0.681 × 0.732, and Reader 2 had ρC2 = 0.573 × 0.656. A statistically significant bias was evident, with MRI-based size estimates being significantly lower than histological measurements. Reader 1 underestimated adenoma size by 15.7% compared to histology (p < 0.001, Sign Rank Test), and Reader 2 underestimated by 18.2% (p < 0.001). Overall, the median maximal lesion diameter measured at surgery was 15 mm (quartiles range 12–20 mm) versus 14 mm (10–19 mm) on MRI (p = 0.026). In SGD patients, adenomas missed by both MRI readers had a median surgical diameter of 18 mm (with quartiles 15–26 mm), like lesions detected by both readers (15 mm with quartiles range12–20 mm; p = 0.19).

## Discussion

This study demonstrates that multiparametric 3T MRI provides high sensitivity for the detection of parathyroid adenomas in patients with SGD, with performance comparable to ultrasonography and MIBI. Sensitivity remained consistently high across two independent readers (0.82 and 0.89), and overall inter-reader reliability was highest for MRI alone (Krippendorff’s α = 0.875), decreasing with the sequential inclusion of ultrasonography and MIBI. Notably, this reduction in agreement occurred despite comparable sensitivities across the individual imaging modalities, suggesting distinct detection profiles of functional and anatomical imaging. Collectively, these findings indicate good reproducibility of MRI interpretation and support its integration into the diagnostic workflow for primary hyperparathyroidism, in line with previously published evidence (9, 10).

Sensitivity was consistently and significantly lower in patients with MGD than in those with SGD across all imaging modalities. Previous studies have shown that negative or discordant imaging is more common in MGD, where findings may falsely suggest SGD (12). In our cohort, MRI correctly identified only 3 of 10 patients with MGD. Lower imaging sensitivity in MGD has been attributed to smaller gland size; however, no significant size difference between MGD and SGD was observed in our study, although MRI-detected lesions in MGD were larger than missed ones. Suppression of smaller or less active glands by dominant adenomas may further reduce detectability. Despite limited imaging sensitivity, surgical outcomes in MGD were excellent, likely reflecting surgical expertise and the use of extended exploration guided by intraoperative findings. Therefore, in patients with discordant or uncertain preoperative parathyroid imaging, surgery was extended to either ipsilateral or bilateral exploration, complemented by perioperative biopsy of resected lesions. Two patients had normocalcemic PHPT which is often associated with multiglandular involvement. Additionally, surgery was extended in four patients with MGD because intraoperative PTH level did not fall significantly after the enlarged parathyroid gland was removed. These findings underscore the challenges of detecting adenomas in MGD, where sensitivity is consistently lower across all imaging modalities. Detection results of a very limited number of MGD highlight the importance of combining functional and anatomical imaging to improve detection and guide surgical planning in patients with MGD, while MRI alone provides robust performance for orthotopic adenomas in SGD.

Although multiparametric MRI with dynamic contrast enhancement was employed in this study, our observations indicated that adenoma detectability relied predominantly on T2-weighted DIXON imaging, whereas the contribution of DCE was perceived by the readers as providing limited additional interpretive value (8). The limited value of DCE was confined largely to differentiating adenomas from thyroid nodules or lymph nodes, yet even in these cases the enhancement patterns showed only minimal distinction. However, this observation was not based on a formal sequence-by-sequence diagnostic performance analysis, and the study was not designed to compare contrast-enhanced and non-contrast MRI protocols. Therefore, no conclusion can be drawn regarding the diagnostic equivalence or sufficiency of a non-contrast MRI protocol.

Missed lesions showed no predilection with respect to size or anatomical location, and no significant difference in maximal lesion diameter was observed between ultrasound and MRI. The median diameter of undetected adenomas was comparable to detected ones, and lesion location proportionally similar. This suggests that the sensitivity of MRI is not substantially affected by these factors which contradicts findings by Bijnens et al. (13). Concordance between readers for adenoma size was good (ρC = 0.822), but agreement with histology was moderate to low (ρC = 0.373–0.498), reflecting systematic underestimation of lesion size by MRI (15–18%). The median lesion size was 15 mm surgically versus 14 mm on MRI, a difference that was statistically significant but likely clinically negligible. Ultrasound measurements were comparable to MRI, with moderate agreement (ρC = 0.694). Observed variability between modalities likely reflects differences in imaging planes and measurement orientation. Overall, these findings support the reliability of MRI and ultrasound for preoperative lesion sizing, while acknowledging limitations in absolute measurement precision.

### Study limitations

Several study limitations shall be acknowledged. The single-center and single vendor design of this study may limit generalizability. Exclusion of examinations with suboptimal MRI quality may have led to overestimation of MRI performance. Ultrasound measurements were dependent on operator experience, and although standardized protocols were used, variability in imaging acquisition and interpretation may have influenced sizing accuracy. The correspondence between locations identified on ultrasound and those found at surgery may be imperfect, as parathyroid glands can shift position with neck tilting. ^18^F-fluorocholine PET/CT was used only in a relatively small number of patients which avoids reliable comparison with MRI. Finally, the study was not designed to compare contrast-enhanced and non-contrast MRI protocols. The perceived incremental value of individual sequences, including dynamic contrast-enhanced imaging, was based on reader interpretation during multiparametric MRI assessment rather than on a separate diagnostic performance analysis of abbreviated protocols.

## Conclusion

MRI demonstrated high sensitivity high sensitivity for localizing parathyroid adenomas in SGD, with performance performance comparable to that of US and SPECT/CT. These findings support the use of multiparametric multiparametric 3-T MRI as a modality for preoperative localization. Preoperative identification of MGD and differentiation between MGD and SGD remain challenging, and MRI does not clearly outperform standard imaging modalities in this setting. Future prospective studies with larger cohorts and predefined sequence-specific analyses or abbreviated-protocol analyses are warranted to determine whether non-contrast MRI protocols can provide diagnostic performance comparable to that of a full contrast-enhanced multiparametric MRI protocol.

## Data Availability

The raw data supporting the conclusions of this article will be made available by the authors, without undue reservation.
