# Supplementary material for: Diagnostic performance of 3T MRI preoperative localization of parathyroid adenomas in primary hyperparathyroidism
Source: Front Endocrinol (Lausanne). 2026 Aug 20;17:1862421. doi: 10.3389/fendo.2026.1862421 (PMC13537962; doi:10.3389/fendo.2026.1862421)
Supplement: Supplementary file 1 [file Table1.docx]

**Electronic supplementary material**

**Suppl. Table 1:** Imaging parameters of MRI sequences

| **Sequence** | **Plane** | **Type** | **TE**  **ms** | **TR**  **s** | **ETL** | **NEX** | **Thickness**  **mm** | **Gap**  **mm** | **Slices** | **Resolution**  **mm** | **Field-of-view**  **mm** | **Time**  **min:s** |
| --- | --- | --- | --- | --- | --- | --- | --- | --- | --- | --- | --- | --- |
| **T2-DIXON** | transverse. | TSE | 100 | 3.0 | 23 | 1 | 3.5 | 0 | 34 | 1.19x1.25 | 190x190x119 | 4:21 |
|  | sagittal. | TSE | 100 | 3.0 | 23 | 1 | 3.5 | 1 | 34 | 1.1x1.16 | 190x190x152 | 4:39 |
|  | coronal | TSE | 100 | 3.0 | 23 | 1 | 3.5 | 1 | 34 | 1.19x1.25 | 190x190x152 | 4:21 |
| **DWIBS** | transverse | DWI STIR | 76 | 7.0 | 83 | 4 | 4 | 0.4 | 40 | 1.9x1.99 | 190x226x176 | 1:59 |
| **DCE^1^** | trasverse | 4D Free breathing | 1.9 | 4.8 | 30 | -1.5 | 3 | -1.5 | 133 | 1.2x1.2 | 370x370x200 | 5:22 |

^1^DCE: dynamic 3D T1-weighted fast field echo with radial stack-of-stars acquistion, 2:00 preparatory scanning and 3:22 dynamic scanning (4,8s x 41 dynamics)

TE – echo time; TR – repetition time; TI – inversion time, ETL – echo train length; TSE – turbo spin echo; DIXON – dual-echo water–fat separation technique; DWI – diffusion-weighted imaging; DCE – dynamic contrast enhancement; NEX – number of excitations
